# Mechanical Stretch Modulates MicroRNA 21 Expression, Participating in Proliferation and Apoptosis in Cultured Human Aortic Smooth Muscle Cells

**DOI:** 10.1371/journal.pone.0047657

**Published:** 2012-10-17

**Authors:** Jian tao Song, Bo Hu, Hai yan Qu, Cheng long Bi, Xiao zhen Huang, Mei Zhang

**Affiliations:** 1 Key Laboratory of Cardiovascular Remodeling and Function Research, Chinese Ministry of Education and Chinese Ministry of Health, Department of Cardiology, Qilu Hospital, Shandong University, Jinan, Shandong, People’s Republic of China; 2 Department of Emergency, Shandong Provincial Hospital, Shandong University, Jinan, Shandong, People’s Republic of China; 3 Department of Cardiology, Shandong Provincial Chest Hospital, Jinan, People’s Republic of China; University of Western Ontario, Canada

## Abstract

**Objectives:**

Stretch affects vascular smooth muscle cell proliferation and apoptosis, and several responsible genes have been proposed. We tested whether the expression of microRNA 21 (miR-21) is modulated by stretch and is involved in stretch-induced proliferation and apoptosis of human aortic smooth muscle cells (HASMCs).

**Methods and Results:**

RT-PCR revealed that elevated stretch (16% elongation, 1 Hz) increased miR-21 expression in cultured HASMCs, and moderate stretch (10% elongation, 1 Hz) decreased the expression. BrdU incorporation assay and cell counting showed miR-21 involved in the proliferation of HASMCs mediated by stretch, likely by regulating the expression of p27 and phosphorylated retinoblastoma protein (p-Rb). FACS analysis revealed that the complex of miR-21 and programmed cell death protein 4 (PDCD4) participated in regulating apoptosis with stretch. Stretch increased the expression of primary miR-21 and pre-miR-21 in HASMCs. Electrophoretic mobility shift assay (EMSA) demonstrated that stretch increased NF-κB and AP-1 activities in HASMCs, and blockade of AP-1 activity by c-jun siRNA significantly suppressed stretch-induced miR-21 expression.

**Conclusions:**

Cyclic stretch modulates miR-21 expression in cultured HASMCs, and miR-21 plays important roles in regulating proliferation and apoptosis mediated by stretch. Stretch upregulates miR-21 expression at least in part at the transcription level and AP-1 is essential for stretch-induced miR-21 expression.

## Introduction

Blood vessels are constantly exposed to mechanical forces in the form of shear stress and cyclic stretch because of pulsatile blood flow. Shear stress is primarily sensed by endothelial cells (ECs), but cyclic stretch can affect all cell types in the vessel wall. Increasing evidence indicates that mechanical forces play critical roles in modulating multiple cellular functions in the vascular wall. Vascular smooth muscle cells (VSMCs), a major constituent of the vessel wall, are the main cell type subjected to cyclic stretch. Under normal conditions, moderate stretch seems essential for maintaining vessel wall structure and vascular homeostasis [1]; however, exacerbated stretch, as in hypertension, could promote pathological vascular remodeling by stimulating VSMC proliferation, apoptosis, migration and abnormal extracellular matrix deposition [2]–[5]. Several biomechanical sensors of stretch (e.g., integrins, ion channels), signaling pathways (e.g., mitogen-activated protein kinases, protein kinase C), and transcription factors (e.g., activating protein 1 [AP-1], nuclear factor κB [NF-κB]) have been implicated [6], but the detailed molecular mechanisms of stretch-induced cellular functions of VSMCs are still not fully elucidated.

MicroRNAs (miRNAs; miRs) are a class of small non-coding RNAs that regulate gene expression at the posttranscriptional level by degrading their target mRNAs or by translational repression [7]. Growing evidence suggests that the alteration in miRNA expression profile plays important roles in the pathogenesis of cardiovascular diseases [8]–[10]. Several studies indicated that miRNAs are also involved in mechanical-force–induced variation in cellular functions *in vitro*. MiRNA expression profile in ECs was found regulated by shear stress, and miR-19a was found to play a critical role in the progression of EC cycle [11]. Stretch upregulated 34 miRNAs and downregulated 8 in rat alveolar epithelial cells [12]. Experimental stretch upregulated the expression of miR-26a, which plays an important role in the regulation of human airway SMC hypertrophy [13]. However, the effect of miRNAs on human aortic SMCs (HASMCs) response to mechanical stretch is still not clear.

MiR-21 is considered an onco-miRNA, its expression was found increased in many solid tumors with characteristics of promoting cell proliferation, migration and anti-apoptosis [14]. MiR-21 is highly expressed in vascular cells and is implicated in vascular disorders such as vascular neointimal lesions [15]. Recently, miR-21 expression in ECs was found regulated by shear stress: laminar shear stress enhanced miR-21 expression, which modulated EC apoptosis and endothelial nitric oxide synthase activity [16], and oscillatory shear stress induced sustained miR-21 expression in ECs, which was implicated in disturbed flow-mediated endothelial inflammation [17].

Many studies have confirmed the effects of stretch on VSMC proliferation and apoptosis, and several candidate genes responsible for these events were proposed; however, whether miR-21 is involved in the regulation of proliferation and/or apoptosis of SMCs mediated by stretch is unclear. In the present study, we investigated whether miR-21 expression in HASMCs is sensitive to mechanical stretch and the underlying mechanism in HASMC proliferation and apoptosis induced by stretch.

**Figure 1:**
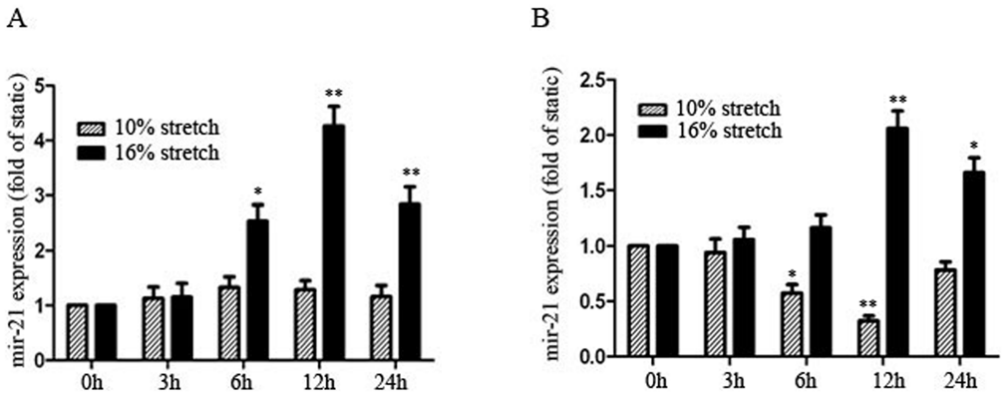
Cyclic stretch modulates miR-21 expression in human aortic smooth muscle cells (HASMCs). Quantitative RT-PCR of level of miR-21 in HASMCs treated with different levels of cyclic stretch (10%, 16% elongation, 1 Hz) for the indicated times (A) without serum and (B) with serum (n = 4, per group). MiR-21 expression was expressed as a ratio to that in static controls (0 h). Data are mean ± SEM, *P<0.05, **P<0.01 vs. static control.

## Methods

### Cell Culture and Cyclic Stretch Application

HASMCs were obtained from ScienCell and were cultured in smooth muscle cell medium (ScienCell, USA) with 5% CO_2_ at 37°C. HASMCs (passages 4 to 7) were seeded (10^5^ cells/well) into 6-well Flexcell plates coated with collagen I, and when they reached 90% confluence, serum-free medium was replaced to induce quiescence for 24 hr. Fresh serum-free medium or complete medium was substituted, then equibiaxial cyclic stretch (10%, 16% elongation, 1 Hz) was applied by use of a computer-controlled Flexcell 5000-Tension apparatus. The apparatus was kept in a humidified incubator with 5% CO_2_ at 37°C. Cells cultured in static conditions were static controls. For inhibition of stretch-induced NF-κB activity, pharmacological inhibitor SN50 was added to the culture medium 1 hr before stretch treatment.

**Figure 2:**
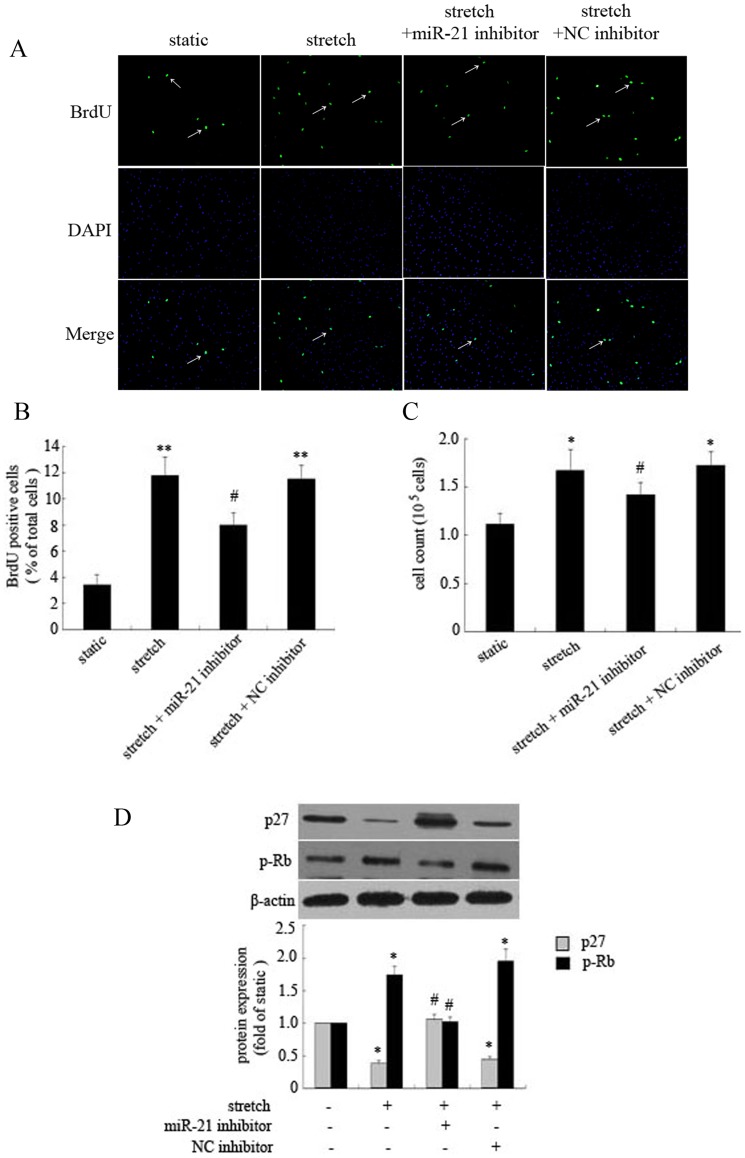
Role of miR-21 in stretch-induced proliferation of HASMCs by regulating p27 and phosphorylated retinoblastoma (p-Rb) protein expression. HASMCs underwent elevated stretch (16% elongation, 1 Hz) or were cultured under static conditions for 12 hr; the effect of stretch on proliferation was determined by BrdU incorporation assay and cell counting. HASMCs were transfected with miR-21 inhibitor or negative control (NC) inhibitor 48 hr before stretch treatment. (A) Representative view of BrdU-positive cells under different conditions, BrdU-positive cells were indicated (white arrows), DAPI was used for nuclear staining. (B) Quantification of BrdU-positive cells. Data are mean ± SEM, n = 4 per group. **P<0.01, versus static control; # P<0.05, versus stretch. (C) Effect of miR-21 on stretch-induced HASMC proliferation determined by cell counting. Data are mean ± SEM, n = 4 per group. *P<0.01, vs. static control; # P<0.01, vs. stretch. (D) Representative western blot and quantification of protein level of p27 and p-Rb. β-actin was a loading control. Data are mean ± SEM, n = 4 per group. *P<0.01, vs. static control; # P<0.01, vs. stretch.

### Quantitative Real-time PCR (qRT-PCR)

After being stretched for the indicated time, HASMCs were harvested, and total RNA, including miRNAs, was extracted by use of TRIzol (Invitrogen, USA); mature miR-21 expression was determined by qRT-PCR by use of the universal cDNA synthesis and SYRB Green Master Mix kits (Exiqon, Denmark), rRNA U6 expression was used as an internal reference for miR-21 expression. The miR-21–specific LNA PCR primer set and primer for U6 were also from Exiqon.

**Figure 3:**
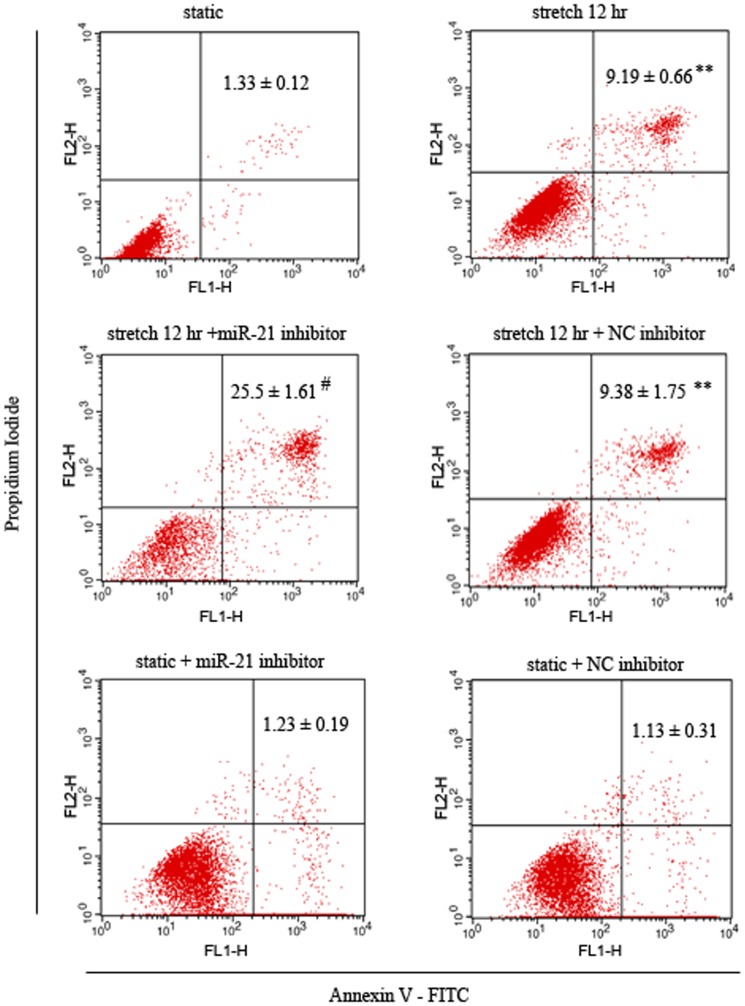
MiR-21 is involved in stretch-induced apoptosis of HASMCs. Representative views of apoptosis of HASMCs under different conditions. HASMCs were cultured under static conditions or underwent elevated stretch (16% elongation, 1 Hz) for 12 hr. MiR-21 or NC inhibitor was added to cells 48 hr before stretch treatment. FACS analysis of Annexin V-FITC and propidium iodide (PI) staining of apoptosis in HASMCs under different conditions. Data are mean ± SEM, n = 4 per group. **P<0.01, vs. static control; # P<0.01, vs. stretch.

**Figure 4:**
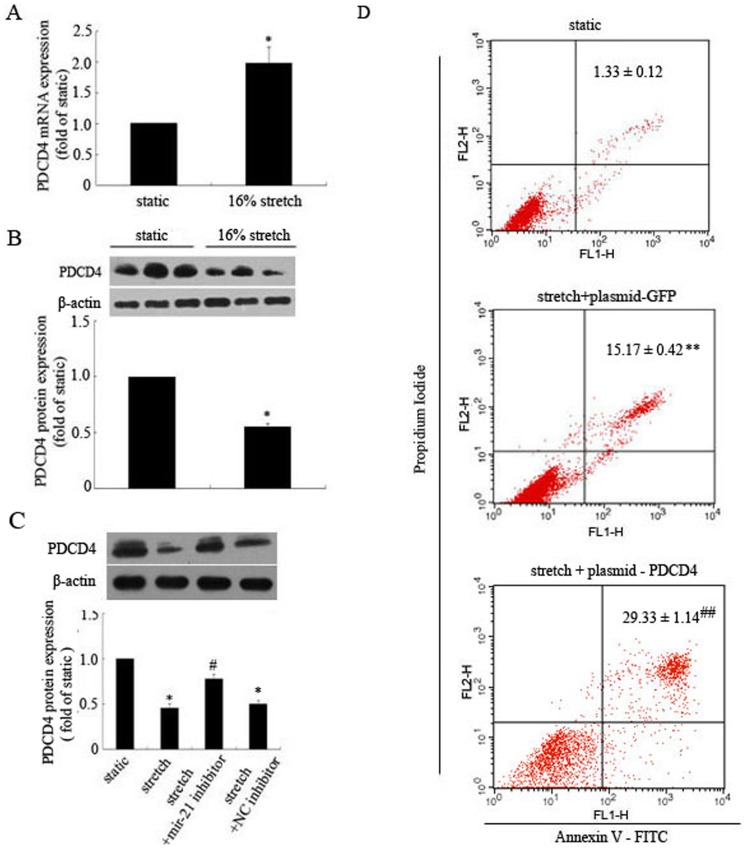
Programmed cell death protein 4 (PDCD4) is a downstream target gene of miR-21 in stretch-induced apoptosis in HASMCs. (A) Quantified RT-PCR analysis of effect of stretch on PDCD4 mRNA expression in HASMCs. HASMCs underwent stretch (16% elongation, 1 Hz) or were maintained under static conditions for 12 hr. Data are mean ± SEM, n = 4 per group. *P<0.01, vs. static control; (B) Western blot analysis of effect of stretch on PDCD4 protein expression in HASMCs. HASMCs underwent stretch (16% elongation, 1 Hz) for 12 hr, or were treated with (C) miR-21 or NC inhibitor after stretch for 12 hr. Data are mean ± SEM, n = 3 per group. *P<0.01, vs. static control; # P<0.05, vs. stretch. (D) Effect of PDCD4 overexpression on apoptosis of stretched HASMCs. HASMCs were transfected with plasmid expressing PDCD4 or GFP, then were exposed to stretch (16% elongation, 1 Hz) for 12 hr. FACS analysis of Annexin V-FITC and PI staining of apoptosis of HASMCs under different conditions. Data are mean ± SEM, n = 4 per group. **P<0.01, vs. static control; ## P<0.01, vs. Stretch + plasmid-GFP.

For detection of the primary miR-21 (pri-miR-21), pre-miR-21 and programmed cell death protein 4 (PDCD4), total RNA was reverse transcribed to cDNA by use of the PrimeScript RT reagent kit (TaKaRa, Japan), and real-time PCR involved use of the SYRB Premix Ex Taq kit (TaKaRa, Japan). PCR cycling conditions were 95°C for 3 min, 40 cycles of 95°C for 10 sec, 56°C for 10 sec, and 72°C for 10 sec. The primer sequences were for pri-miR-21, forward, 5′-TTTTGTTTTGCTTGGGAGGA-3′ and reverse, 5′-AGCAGACAGTCAGGCAGGAT-3′; pre-miR-21, forward, 5′-TGTCGGGTAGCTTATCAGAC-3′ and reverse, 5′-TGTCAGACAGCCCATCGACT-3′; PDCD4, forward, 5′-TGAGCACGGAGATACGAACGA-3′ and reverse, GCTAAGGACACTGCCAACACG-3′; and β-actin, forward, 5′-CGTGCGTGACATTAAGGAGA-3′ and reverse, 5′-CACCTTCACCGTTCCAGTTT-3′. β-actin was a internal reference of pri-miR-21, pre-miR-21 and PDCD4 levels [18].

**Figure 5:**
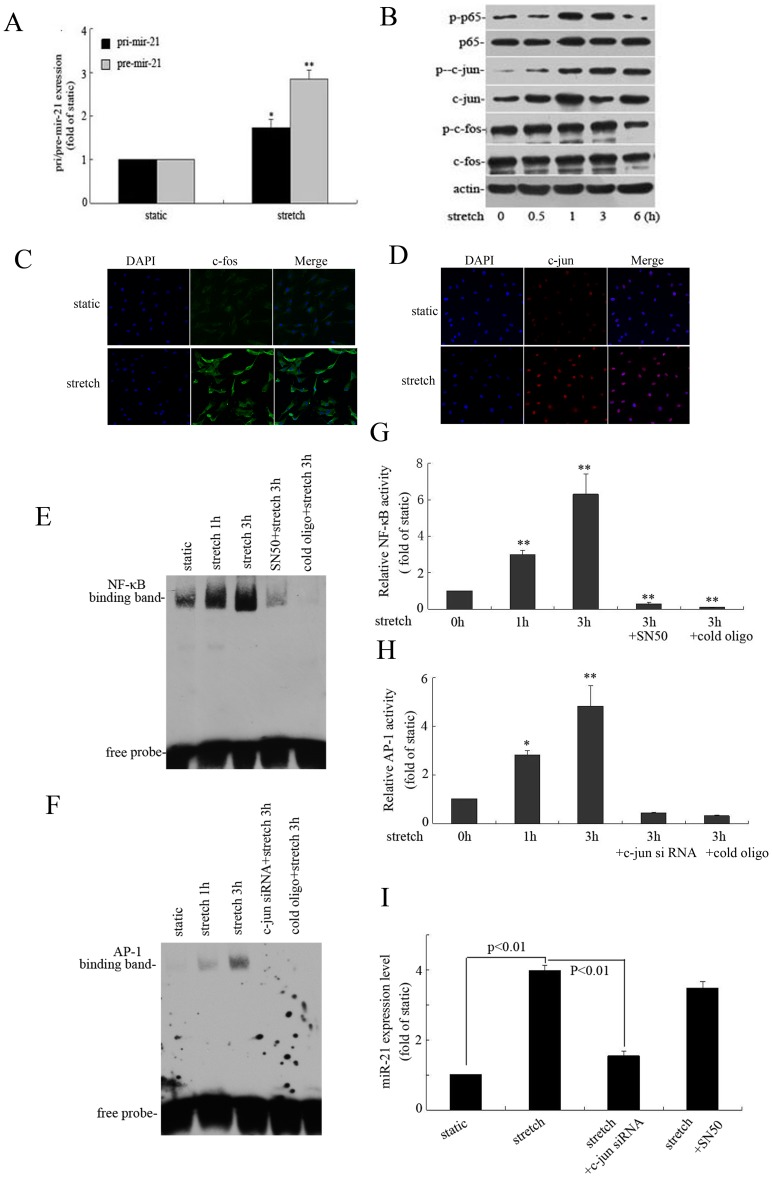
Transcription factor activating protein 1 (AP-1; c-jun) is responsible for elevated stretch-induced miR-21 expression in HASMCs. (A) Quantitative RT-PCR analysis of effect of stretch on primary miR-21 (pri-miR-21) and pre-miR-21 expression in HASMCs that underwent stretch (16% elongation, 1 Hz) or were cultured under static conditions for 12 hr. Data are mean ± SEM, n = 4 per group. *P<0.05, **P<0.01, vs. static control. (B) Representative western blot analysis of protein levels of p65, c-jun, c-fos and their phosphorylation in HASMCs subjected to stretch (16% elongation, 1 Hz) for the indicated times. (C) and (D) Stretch induced c-jun and c-fos expression. HASMCs were exposed to stretch for 3 hr, the immunofluorescence staining was performed using anti-c-jun and anti-c-fos antibodies, the slides were counterstained with DAPI, Images were obtained using laser scanning confocal microscopy. (E) and (F) Representative images of EMSA for detecting NF-κB and AP-1 DNA binding activities in HASMCs under different conditions. HASMCs were exposed to stretch (16% elongation, 1 Hz) for 1 and 3 hr, Pharmacological inhibitor for NF-κB (SN50) were added 1 hr before stretch treatment, c-jun or scramble siRNA was introduced into cells 48 hr before stretch treatment. (G) and (H) Quantitative analysis of NF-κB and AP-1 activities in HASMCs under different conditions. Data are mean ± SEM, n = 3 per group. *P<0.05, **P<0.01 vs static control (0 h). (I) Effect of c-jun siRNA and SN50 on miR-21 expression induced by stretch (16% elongation, 1 Hz) in HASMCs. Data are mean ± SEM, n = 4 per group.

### Western Blot Analysis

Protein from HASMCs stretched or cultured in static conditions was extracted by use of a cytoplasmic extraction reagent kit (Pierce, USA). Protein concentrations were determined by the BCA method. In brief, 15–20 µg protein extract was separated by SDS-PAGE and transferred to PVDF membrane (Millipore, USA), which was blocked with 5% nonfat milk for 2 hr. Then membranes were incubated with primary antibodies overnight at 4°C. After a washing with 1×TBST 3 times, membranes were incubated with secondary antibodies for 1 hr. Visualization involved an enhanced chemiluminescence-plus detection system (Millipore). Relative band intensities were analyzed by use of Photoshop CS3.

### Transient Transfection

For miR-21 inhibition and overexpression, miR-21 inhibitor and mimics were synthesized by GenePharm (Shanghai, China). The sequence for miR-21 inhibitor was: 5′-UCAACAUCAGUCUGAUAAGCUA-3′; the sequences for miR-21 mimics were: 5′-UAGCUUAUCAGACUGAUGUUGA-3′ and 5′- AACAUCAGUCUGAUAAGCUAUU-3′; the sequence for the negative control (NC) miRNA inhibitor was: 5′-CAGUACUUUUGUGUAGUACAA-3′. The miR-21 inhibitor or mimics was transfected into HASMCs by use of Lipo2000 (Invitrogen) at a final concentration of 90 nM 48 hr before stretch treatment.

For downregulation of c-jun expression, c-jun siRNA and negative control (NC) siRNA were synthesized by GenePharm (Shanghai, China). The sequences for c-jun siRNA were: 5′-CCAAGAACUGGACAGAUGA-3′ and 5′-UCAUCUGUCACGUUCUUGG-3′; the sequences for NC siRNA were: 5′-UUCUCCGAACGUGUCACGUTT-3′ and 5′-ACGUGACACGUUCGGAGAATT-3′. SiRNAs for c-jun or NC was transfected into HASMCs using Lipo2000 (Invitrogen) at a final concentration of 60 nM 48 hr before stretch treatment.

For PDCD4 overexpression, a plasmid expressing PDCD4 (gift from Professor LN Zhang, Institute of Immunology, Shandong university, Jinan, China) was transfected into HASMCs by use of Lipo2000 (Invitrogen) 48 hr before stretch; a plasmid expressing green fluorescent protein (GFP) was used as a negative control. The transfection medium was replaced 4 hr after transfection by fresh serum-free medium.

### Cell Proliferation Analysis

Cell proliferation was determined by cell counting and BrdU incorporation. HASMCs were exposed to stretch or maintained in static conditions for 12 hr, then the medium was removed and cells were washed with cold phosphate buffered saline twice. Cells were harvested by trypsinization and were counted (Beckman Coulter, USA). BrdU incorporation assay was performed according to the manufacturer’s instructions (Roche Applied Science, Indianapolis, IN, USA).

### Annexin V and Propidium Iodide (PI) Double Staining and FACS Analysis

We used the Annexin V-FITC Apoptosis Detection Kit (BIPEC, USA) to determine apoptotic cells. In brief, cells with or without stretch treatment were resuspended with 400 µl binding buffer at 10^6^ cells/ml, underwent 15-min annexin V-FITC labeling, then 5-min PI labeling, then were analysed by flow cytometry in 1 hr. In total, 10,000 cells were counted in each assay.

### Immunofluorescence Staining

Briefly, HASMCs were fixed with Immunol Staining Fix Solution (Beyotime, Shanghai, China) for 30 min at room temperature. Cells were treated with PBS containing 0.1% Triton X-100 for 8 min and blocked by incubating in 5% bovine serum albumin for 30 min. After washing with PBS, cells were incubated with c-jun or c-fos primary antibodies (Cell Signaling Technology, 1∶200) overnight at 4°C. After washing 3 times with PBS containing 0.5% TritonX-100, the cells were stained with Fluor secondary antibodies for 1 hr, then DAPI for 3 min at room temperature. Images were obtained using laser scanning confocal microscopy (LSM710, ZEISS, Germany).

### Electrophoretic Mobility Shift Assay (EMSA)

To determine the effect of stretch on AP-1 and NF-κB activity in HASMCs, EMSA was performed. In brief, nuclear protein was extracted from HASMCs subjected to cyclic stretch or not by use of a nuclear protein kit (Pierce, USA), and protein concentrations were determined by the BCA method. Double-stranded gel-shift oligonucleotides for AP-1 and NF-κB were end-labeled with [γ^−32^P] ATP with T4 kinase. The nuclear extracts (8 µg) were mixed with labeled oligonucleotides for AP-1 or NF-κB and other important components in a total volume of 20 µl for 30 min, then separated by 4% nondenaturing acrylamide PAGE. The detailed procedures followed the manufacturers’ instructions of EMSA kit (Pierce, Thermo Scientific, USA).

### Statistical Analysis

All data are expressed as mean±SEM. Statistical significance was determined by one-way ANOVA or two-tailed unpaired Student’s *t* test, *P*<0.05 was considered statistically significant.

## Results

### Mechanical Stretch Modulates miR-21 Expression in HASMCs

HASMCs were exposed to cyclic stretch (10%, 16% elongation, 1 Hz) with or without serum for the indicated times. Only elevated stretch (16% elongation, 1 Hz) significantly increased miR-21 expression as compared with the static control in the absence of serum (Fig. 1A); the peak increase was at 12 hr and was maintained at 24 hr. Previous studies indicated that physiological moderate stretch inhibited growth-factor–induced proliferation, whereas enhanced stretch seemed to have opposite effects on proliferation of VSMCs [1], [5]. We further investigated the combined effects of stretch and serum on miR-21 expression in cultured HASMCs. With serum, moderate stretch significantly reduced miR-21 expression as early as 6 hr, which was sustained for 24 hr, whereas elevated stretch (16% elongation, 1 Hz) increased miR-21 expression at 12 hr, which was maintained at a high level at 24 hr (Fig. 1B).

### Increased miR-21 Expression is Involved in Elevated Stretch-induced HASMCs Proliferation

MiR-21 level was found related to proliferation of cultured rat VSMCs, and several studies also demonstrated that elevated stretch promotes VSMCs proliferation [19], [20], [21]. To identify the role of increased miR-21 in regulating HASMCs proliferation mediated by stretch, cells were transfected with miR-21 or NC inhibitor, and the effect of stretch on HASMCs proliferation was determined by BrdU incorporation assay and cell counting. BrdU-positive cells were increased significantly in number after elevated stretch (16% elongation, 1 Hz) treatment for 12 hr as compared with the static controls; however, the response was suppressed with miR-21 inhibition (Fig. 2A, B). Data from cell counting were consistent with BrdU incorporation results (Fig. 2C). To further explore the molecular mechanisms mediating the effect of miR-21 on proliferation in stretched HASMCs, we determined the protein level of p27 and p-Rb in HASMCs subjected to elevated stretch or static conditions. Stretch significantly reduced p27 level and increased p-Rb expression in HASMCs; these effects were partly abolished by miR-21 inhibition. Thus, increased miR-21 expression was involved in elevated-stretch–induced HASMC proliferation, likely by regulating p27 and p-Rb expression.

### MiR-21 Participates in Regulating Elevated Stretch-induced HASMC Apoptosis

MiR-21 was found upregulated in various solid tumors and associated with anti-apoptosis or pro-survival effects [14]. We verified whether miR-21 is involved in stretch-induced apoptosis of HASMCs by FACS analysis of Annexin V–PI double staining of HASMCs exposed to elevated stretch (16% elongation, 1 Hz) or static conditions. As shown in Fig. 3, elevated stretch slightly increased the apoptosis of stretched HASMCs as compared with the static controls; miR-21 inhibitor treatment before stretch significantly increased apoptosis of HASMCs, but the NC inhibitor had no effect. Of interest, miR-21 inhibition had no effect on apoptosis of HASMCs under static conditions. Therefore, increased miR-21 expression by elevated stretch may help inhibit stretch-induced apoptosis of HASMCs.

### PDCD4 is a Downstream Target of miR-21 Responsible for Elevated Stretch-induced Apoptosis of HASMCs

PDCD4 plays an important role in regulating apoptosis of many types of cells and is a direct target gene of miR-21 [22], [23]. To verify whether PDCD4 is involved in apoptosis of HASMCs mediated by stretch, we observed the effects of elevated stretch (16% elongation, 1 Hz) on the mRNA and protein levels of PDCD4. Stretch significantly induced PDCD4 mRNA expression (1.98-fold), but the protein level was downregulated as compared with the static control (0.55-fold) (Fig. 4A, B). Therefore, stretch regulated PDCD4 expression at the post-transcriptional level. In addition, inhibition of miR-21 expression abolished the inhibitory effect of stretch on PDCD4 protein level (Fig. 4C). To further identify the role of PDCD4 in stretch-induced apoptosis of cells. HASMCs transfected with plasmid-PDCD4 were exposed to stretch, apoptosis was greater than GFP (Fig. 4D). Therefore, PDCD4 may be a downstream target of miR-21 responsible for antagonizing stretch-induced apoptosis of HASMCs.

### AP-1 (c-jun) is Responsible for Elevated Stretch-induced miR-21 Expression in HASMCs

Many studies have focused on the cellular functions of miR-21 in various physiological or pathological conditions, but little is known about the regulation of miR-21 expression. To explore the mechanism underlying stretch-induced miR-21 expression, we determined pri-miR-21 and pre-miR-21 expression. After 12-hr stretch (16% elongation, 1 Hz), pri-miR-21 expression was increased 1.73-fold and pre-miR-21 expression 2.85-fold as compared with the static control (Fig. 5A). AP-1 and NF-κB are important transcription factors involved in stretch mediated cellular functions of VSMCs. In our study, the phosphorylation of c-jun and p65 in the nucleus was increased significantly with stretch; however, that of c-fos was not affected (Fig. 5B). Besides, results from Immunofluorescence revealed that stretch significantly increased c-jun expression in the nucleus, however, increased c-fos expression in cytoplasm, as shown in Fig.5C and 5D. The experimental data of EMSA showed that stretch obviously increased NF-κB and AP-1 DNA binding activities and SN50, c-jun siRNA could abolished their activation induced by stretch (Fig. 5E-H). Previous researches suggested that miR-21 expression may depend on AP-1 or NF-κB [17], [24]. We pretreated cells with SN50 and c-jun siRNA to block their activation induced by stretch. We found that c-jun siRNA significantly attenuated the stretch-induced increase in miR-21 expression, but SN50 had no effect (Fig. 5I). Thus, AP-1 (c-jun) may be involved in stretch-induced miR-21 expression.

## Discussion

Here, we demonstrated that elevated stretch enhanced miR-21 expression in HASMCs with or without serum stimulation, but moderate stretch suppressed miR-21 expression only with serum stimulation. Increased miR-21 expression may be involved in the regulation of HASMC proliferation and apoptosis mediated by stretch mainly through AP-1.

MiRNAs are a class of small non-coding RNA that negatively regulate target genes at the posttranscriptional level, a large body of studies revealed that miRNAs almost participate in all cellular functions [25], [26], [27]. Recently, several researches indicated that mechanical force, both shear stress and cyclic stretch, could modulate the expression of miR-21, mir-19a, mir-23b, and mir-26a in cultured cells [11], [13], [16], [28]. These miRNAs are involved in the cellular response to mechanical forces. MiR-21 was found upregulated in many types of solid tumors and cardiovascular disorders [14], [19], [29]. In the present study, we demonstrated that stretch could modulate miR-21 expression in HASMCs: elevated stretch (16% elongation, 1 Hz) induced a sustained increase in miR-21 expression, whereas moderate stretch (10% elongation, 1 Hz) had no effect. Furthermore, we investigated the combined effects of serum stimulation and levels of stretch on miR-21 expression in cultured HASMCs: moderate stretch repressed miR-21 expression but elevated stretch still induced miR-21 expression. These results suggest that the effects of stretch on miR-21 expression depend on the stretch magnitude, and culture conditions influence miR-21 levels regulated by stretch. Our results are consistent with those of several studies. MiR-21 was found upregulated by steady laminar shear stress in ECs [16]; its expression was inhibited by pulsatile shear stress but induced by oscillatory shear stress [17]. Therefore, miR-21 levels in vascular cells are sensitive to variations of mechanical forces.

VSMCs exposed to elevated stretch showed increased proliferation and apoptosis, but the molecular mechanisms involved in the processes are not clearly understood. Inhibition of miR-21 expression in cultured rat VSMCs significantly reduced cell proliferation, and downregulation of elevated miR-21 level in the injured carotid artery notably decreased neointimal formation [19]. However, whether miR-21 is involved in stretch-mediated cellular functions of VSMCs is not clear. In our study, we firstly determined the effect of miR-21 on proliferation of HASMCs mediated by stretch. Elevated stretch significantly induced HASMC proliferation. With miR-21 inhibition, stretch-induced proliferation of HASMCs was partially abolished, as demonstrated by BrdU incorporation and cell counting. Actually, we also investigated the role of miR-21 on proliferation of HASMCs under moderate stretch (10% elongation, 1 Hz), and our results suggested that overexpression of miR-21 partially reversed the inhibitory effect of moderate stretch on HASMCs proliferation (supporting information). So, these results indicated miR-21 is implicated in the regulation of proliferation by stretch. Elevated stretch could induce p27 downregulation via a PI3K/Akt pathway, which led to cell cycle entry and progression [20]. We also found that elevated stretch significantly repressed p27 expression and increased p-Rb level, but these effects were partially abolished with miR-21 inhibition. Therefore, increased miR-21 expression contributed to stretch-induced cell proliferation by reducing p27 expression and increasing p-Rb expression; however, the detailed mechanisms still need further investigation because p27 is not a direct target of miR-21. Previous studies have confirmed that PTEN, a negative regulator of PI3K/Akt pathway, is a direct target of miR-21 [16], [19]. The elevated miR-21 level could suppress PTEN expression, thus leading to the activation of PI3K/Akt pathway, which may be a potential mechanism for miR-21–mediated p27 expression.

The balance between cell proliferation and apoptosis plays a critical role in vascular remodeling. The effects of stretch on apoptosis in cultured VSMCs are contradictory; different magnitudes of applied stretch, duration, or cell species may explain the conflicting results [30], [31], [32]. In our study, we examined the effects of elevated stretch on apoptosis of HASMCs, and FACS analysis demonstrated only slightly increased apoptosis after stretch treatment. However, HASMCs with miR-21 inhibition significantly increased apoptosis as compared with those without miR-21 inhibition after stretch treatment. Of note, inhibition of miR-21 in HASMCs under static conditions had no effect on apoptosis. Thus, increased miR-21 expression may be a negative regulator of stretch-induced apoptosis.

PDCD4 is a verified target gene of miR-21 and is involved in regulating apoptosis of many types of cells. Overexpression of PDCD4 by adenovirus significantly promoted rat VSMCs apoptosis *in vitro*, and inhibition of PDCD4 was related to decreased apoptosis of cells [22]. To determine the role of PDCD4 in stretch-induced apoptosis of HASMCs, we examined the effects of stretch on PDCD4 expression at mRNA and protein levels. Elevated stretch increased PDCD4 mRNA expression but significantly reduced PDCD4 protein expression. Therefore, stretch-regulated PDCD4 expression mainly occurs at the posttranscriptional level. Furthermore, inhibition of miR-21 in stretched HASMCs increased PDCD4 protein expression, and overexpression of PDCD4 in stretched HASMCs showed a significant increased apoptosis. Therefore, stretch-enhanced miR-21 expression plays an important role in promoting HASMC survival by regulating PDCD4 expression.

So far, studies mainly focused on the biological effects of miRNAs, but little is known about the regulatory mechanisms of miRNAs under various pathological or physiological conditions. Recently, several studies indicated that miRNA levels can be regulated at transcriptional or posttranscriptional level [18], [33], [34]. We investigated the effect of stretch on pri-miR-21 and pre-miR-21 levels and found that it significantly increased pri-miR-21 expression (1.73-fold vs. the static control), which indicates that stretch-induced miR-21 expression occurs at least in part at the transcriptional level. The pre-miR-21 level was also increased obviously by stretch stimulation (2.85-fold vs. the static control). Of note, the stretch-increased expression of pre-miR-21 was much higher than that of pri-miR-21, so stretch-induced miR-21 expression may also occur at the posttranscriptional level.

AP-1and NF-κB are transcription factors often activated by stretch, participating the regulation of proliferation and apoptosis. Several studies reported that AP-1 and NF-κB were involved in the regulation of miR-21. Oscillatory shear stress induced miR-21 expression in ECs through AP-1 [17] and lipopolysaccharide enhanced miR-21 expression in human biliary epithelial cells through NF-κB p65 subunit binding to the promoter elements of miR-21 [35]. We found the expression of p-p65 and p-c-jun, as well as NF-κB and AP-1 DNA binding activities, increased with elevated stretch. To test whether the 2 transcription factors are involved in stretch-induced miR-21 expression in cultured HASMCs, we used SN50 and c-jun siRNA to block their activation. We found that siRNA for c-jun significantly suppressed the stretch-upregulated expression of miR-21, but SN50 had no effect. Therefore, AP-1 (c-jun) may be responsible for miR-21 expression mediated by elevated stretch.

In summary, miR-21 may be involved in the regulation of proliferation and apoptosis mediated by mechanical stretch in HASMCs; p27 and PDCD4 may be the downstream target genes responsible for miR-21–mediated cellular functions in stretched cells; and the increased miR-21 expression by stretch may at least occur at the transcriptional level through AP-1. Our findings suggest a potential target for treating human vascular disorders related to elevated mechanical stretch, such as hypertension and atherosclerosis.

## Supporting Information

Figure S1 Role of miR-21 in modest 10% stretch induced inhibition of HASMCs proliferation. BrdU staining was performed to identify the proliferation of HASMCs, we counted 3 different views under 100×magnifications. (A) Representative images of BrdU-positive cells under different conditions, BrdU-positive cells were indicated (white arrows), DAPI was used for nuclear staining. HASMCs were exposed to modest stretch (10% elongation, 1 Hz) or maintained in static conditions for 12 hr in the presence of serum; miR-21 or scramble mimics was introduced into cells 48 hr before modest 10% stretch treatment. (B) Quantification of BrdU-positive cells for different conditions, data are expressed as mean±SEM, n = 4.
(TIF)

Figure S2 Effect of modest 10% stretch on apoptosis of HASMCs. Cultured HASMCs were exposed to modest stretch (10% elongation, 1 Hz) or maintained at static conditions for 12 hr, then Annexin V-FITC and propidium iodide (PI) staining was performed. FACS analysis was used to evaluate the effect of 10% stretch on apoptosis of HASMCs. Data are expressed as mean±SEM, n = 4.
(TIF)
