# Supplementary figures and images for: Mechanical Stretch Modulates MicroRNA 21 Expression, Participating in Proliferation and Apoptosis in Cultured Human Aortic Smooth Muscle Cells
Source: PLoS One. 2012 Oct 17;7(10):e47657. doi: 10.1371/journal.pone.0047657 (PMC3474731; doi:10.1371/journal.pone.0047657)

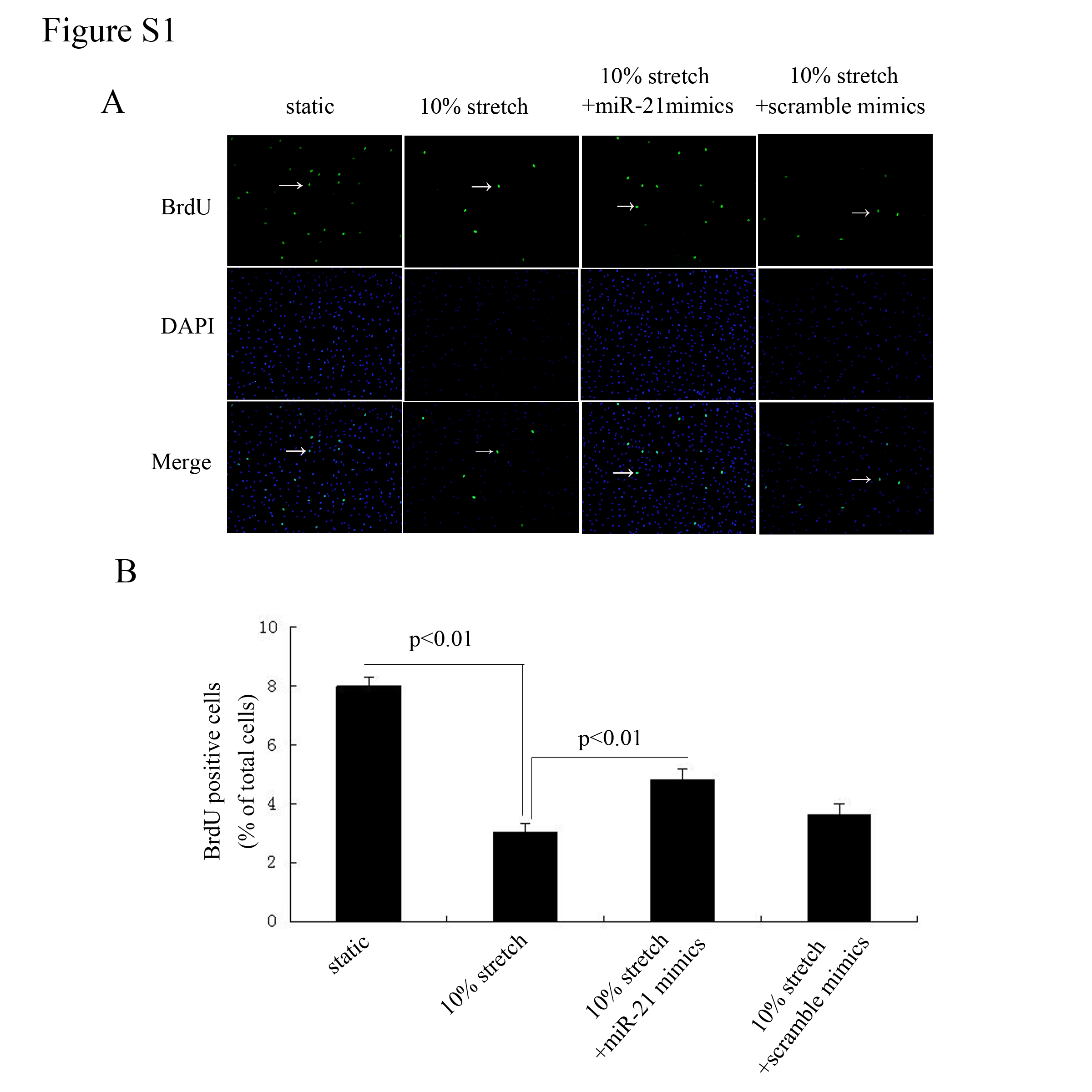

Supplement: Figure S1 — Role of miR-21 in modest 10% stretch induced inhibition of HASMCs proliferation. BrdU staining was performed to identify the proliferation of HASMCs, we counted 3 different views under 100×magnifications. (A) Representative images of BrdU-positive cells under different conditions, BrdU-positive cells were indicated (white arrows), DAPI was used for nuclear staining. HASMCs were exposed to modest stretch (10% elongation, 1 Hz) or maintained in static conditions for 12 hr in the presence of serum; miR-21 or scramble mimics was introduced into cells 48 hr before modest 10% stretch treatment. (B) Quantification of BrdU-positive cells for different conditions, data are expressed as mean±SEM, n = 4. (TIF) [file pone.0047657.s001.tif]

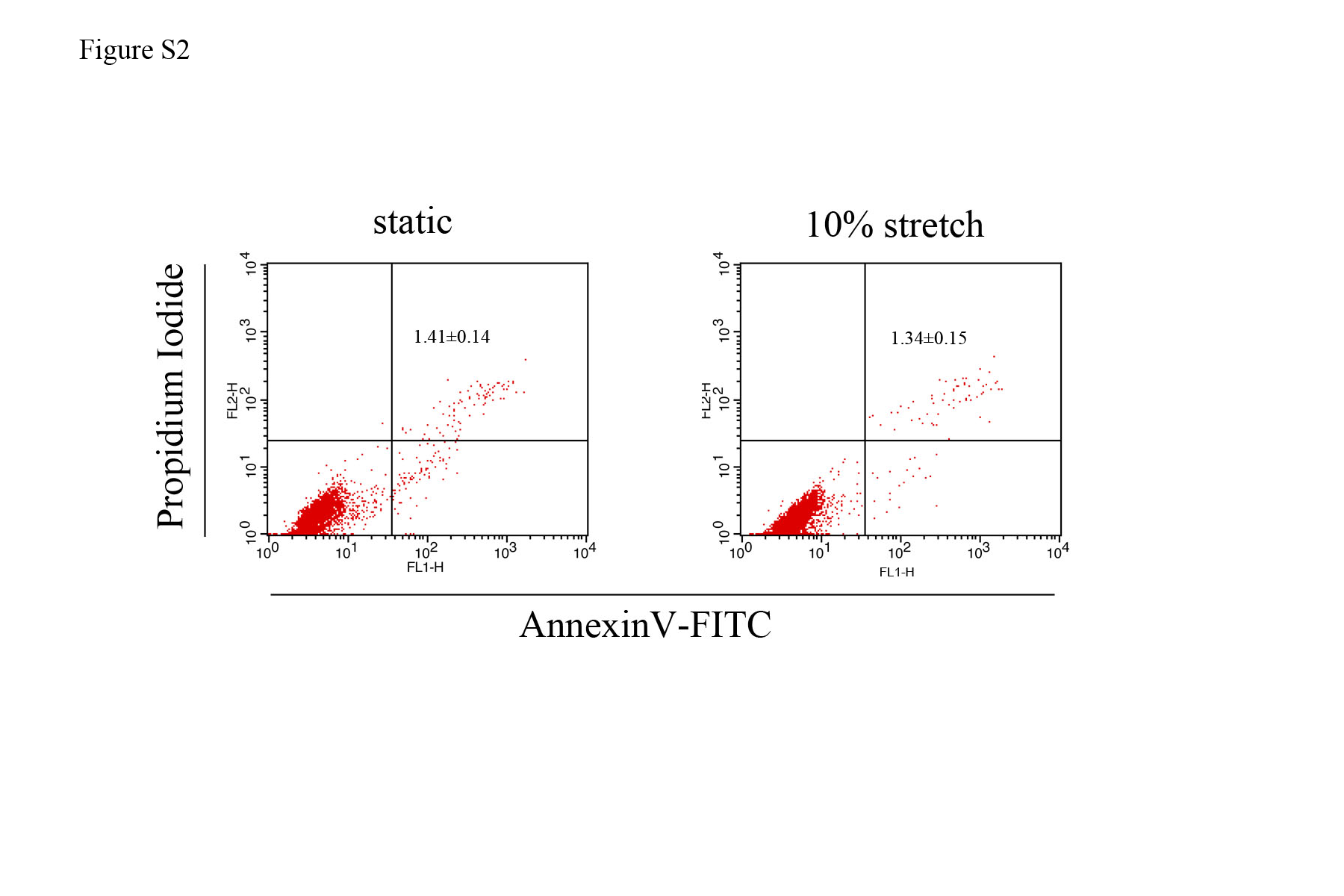

Supplement: Figure S2 — Effect of modest 10% stretch on apoptosis of HASMCs. Cultured HASMCs were exposed to modest stretch (10% elongation, 1 Hz) or maintained at static conditions for 12 hr, then Annexin V-FITC and propidium iodide (PI) staining was performed. FACS analysis was used to evaluate the effect of 10% stretch on apoptosis of HASMCs. Data are expressed as mean±SEM, n = 4. (TIF) [file pone.0047657.s002.tif]
